# Shared genetic architecture of HCM and AF: Elevated *NPPA*/ANP is linked to atrial electrical remodeling

**DOI:** 10.1371/journal.pone.0357969

**Published:** 2026-09-24

**Authors:** A. Z. M. Fahim Siddique, Xinru Wang, Ruyi Du, Yuchen Wang, Qi Wang, Yuanyuan Guo, Tiankai Li

**Affiliations:** 1 Department of Cardiology, The First Affiliated Hospital of Harbin Medical University, Harbin, China; 2 Department of Cardiology, Beidahuang Industry Group General Hospital, Harbin, China; 3 Department of Geriatrics, The First Affiliated Hospital of Harbin Medical University, Harbin, China; University of Campania, ITALY

## Abstract

**Background:**

Hypertrophic cardiomyopathy (HCM) patients exhibit heightened susceptibility to atrial fibrillation (AF), yet the underlying genetic nexus remains poorly defined. This study aimed to identify genes common to HCM and AF, thereby revealing the underlying pathogenesis of susceptibility to AF in HCM patients.

**Methods:**

Genes associated with HCM and AF were sourced from the Gene Expression Omnibus (GEO), Online Mendelian Inheritance in Man (OMIM), Comparative Toxicogenomics Database (CTD), GeneCards, and DisGeNET databases. We conducted a comparative analysis to identify overlapping genes between HCM and AF for further investigation, which included functional enrichment analysis, protein-protein interaction networks, and immune infiltration assessment. Key hub genes were determined using cytoHubba, a Cytoscape plugin, and their validation was performed using independent datasets GSE36961 and GSE115574. The genes corroborated by these datasets were additionally validated in clinical samples.

**Results:**

A total of 11 overlapping genes were identified between HCM and AF. Functional enrichment and immune infiltration analyses revealed their involvement in immune regulation and inflammatory processes. Three hub genes *NPPA*, *ACE*, and *POMC* were recognized as key molecular links between HCM and AF. Validation using the GSE36961 dataset confirmed the upregulation of *NPPA* in HCM, supporting its potential as a central gene connecting the two conditions. Consistent with the bioinformatics findings, exploratory clinical verification findings of serum levels of N-terminal fragment of A-type natriuretic peptide (NT-proANP), encoded by *NPPA*, were markedly elevated in both the HCM and HCM with AF groups compared with healthy controls (*P* < 0.01). Moreover, NT-proANP concentrations were significantly higher in the HCM + AF group than in the HCM-only group (*P* < 0.01).

**Conclusion:**

This study identifies a shared genetic link between HCM and AF. The findings strongly associate elevated NT-proANP levels, encoded by the *NPPA* gene, with AF in HCM patients. We propose NT-proANP as a promising circulating biomarker for stratifying AF risk in this patient population.

## Introduction

Hypertrophic cardiomyopathy (HCM) is a genetic cardiac disease characterized by abnormal thickening of the myocardial tissue, which can result in various complications, including heart failure (HF), arrhythmias, and sudden cardiac death. Among these complications, atrial fibrillation (AF) is the most common, affecting approximately 25% of HCM patients [1,2]. Patients with HCM are estimated to have a 4–6-fold increased likelihood of developing AF over their lifetime [3,4]. The interplay between HCM and AF is complex, involving structural and electrical remodeling of the heart that heightens the risk of adverse outcomes such as stroke and heart failure [5].

Hemodynamic abnormalities, including diastolic dysfunction and left ventricular outflow tract obstruction (LVOTO), are considered key factors in the progressive enlargement of the left atrium (LA) and the subsequent onset of AF in HCM. An increase in LA volume correlates with a higher prevalence of AF in HCM [6]. An LA strain of < 23.4% is recognized as an independent risk factor alongside LA volume, and both LA size and contractile function have been found to predict new-onset AF [7]. Mitral regurgitation, LVOTO, and increased ventricular filling pressures are the main causes of the typical LA structural remodeling in HCM. Furthermore, it is common to see atrial fibrosis, which is caused by ischemia and microvascular dysfunction and leads to additional atrial dilatation and mechanical impairment [8]. When combined, these changes produce a substrate that promotes the development of AF.

The majority of prior studies on AF in HCM have been descriptive, concentrating on clinical occurrence, prognosis, and imaging characteristics. The biochemical and genetic processes underlying AF susceptibility in this population, however, have not been extensively studied [9]. By combining extensive genetic data with bioinformatics-based analyses, these pathways can be investigated. Therefore, this study aimed to identify the shared genetic architecture between HCM and AF and to investigate molecular links that could inform biomarker discovery for AF risk in HCM patients.

## Materials and methods

### Acquisition of HCM and AF gene datasets

We conducted a systematic search of various disease-associated databases, including Online Mendelian Inheritance in Man (OMIM) (http://omim.org/), Comparative Toxicogenomics Database (CTD) (http://ctdbase.org/), GeneCards (https://www.genecards.org/), and DisGeNET (https://www.disgenet.org/), to identify genes associated with HCM and AF. Simultaneously, transcriptomic datasets were obtained from the Gene Expression Omnibus (GEO) database. Three datasets were chosen: GSE180313, GSE41177, and GSE79768, which include expression profiles from 27 patients with HCM and 13 controls, as well as from 29 patients with AF and 19 non-AF individuals.

For databases that provide a quantitative metric of gene-disease association (e.g., GeneCards’ Relevance score’, DisGeNET ‘score’), the ‘relevant genes’ were defined as the top 500 genes when sorted in descending order of that score. If a database listed fewer than 500 genes for a given disease, all available genes were included. For databases without a quantitative scoring system (e.g., OMIM), all curated genes for the disease were collected. This process yielded initial gene sets for HCM and AF from each source. Differentially expressed genes (DEGs) were identified from the GEO datasets utilizing the DESeq2 package in R, with a threshold of |log_2_FoldChange| > 1 and a false discovery rate (FDR) < 0.05. The genes associated with HCM and AF were generated by integrating differentially expressed genes with those from disease databases. The overlap of HCM- and AF-related gene sets was utilized to identify common genes for subsequent analyses. Data access and retrieval occurred from August 25 to September 2, 2025.

### Profiling of functional and pathway enrichment

Biological implications of the overlapping gene set were examined using Gene Ontology (GO) and Kyoto Encyclopedia of Genes and Genomes (KEGG) enrichment analyses with the clusterProfiler package in R. Enrichment terms were considered significant at *P* < 0.05. The analyses provided a comprehensive understanding of the biological processes, cellular components, molecular functions, and signalling pathways most prominently associated with the shared genes.

### Gene enrichment analysis

GO and KEGG enrichment analyses were used to investigate the biological activities and signaling pathways associated with the common genes between HCM and AF. Analyses were performed using the cluster Profiler package in R, with a significance threshold of *P* < 0.05. These studies enabled us to classify biological processes, molecular functions, and cellular components, and to uncover key metabolic and signaling pathways enriched among overlapping genes.

### Protein–Protein Interaction (PPI) network analysis and screening for common hub genes

Using the STRING database (https://string-db.org/) and a medium confidence interaction score of more than 0.4, we created a protein-protein interaction (PPI) network. Cytoscape software (version 3.9.1) was used to visualize the PPI network [10]. Five topological algorithms: BottleNeck, Closeness, Radiality, Betweenness, and Stress in the CytoHubba plugin of Cytoscape to identify the key hub genes. The intersection of the top 5 genes from each algorithm was defined as the set of common hub genes associated with both HCM and AF.

### Immune microenvironment profiling and hub gene correlation analysis

The immune cell composition of the HCM and AF samples was analyzed using CIBERSORTx, a deconvolution algorithm that estimates the relative fractions of immune cell types based on gene expression profiles [11]. The immune infiltration landscape was quantified for each sample, and the relationships between hub gene expression levels and individual immune cell populations were evaluated using the Sangerbox online platform [12].

### Validation of hub genes in external datasets

To validate the discovered hub genes and reduce false positives, separate datasets from the GEO database were employed. HCM validation was performed using the dataset GSE36961, which included transcriptome data from 39 non-HCM controls and 106 HCM patients. For AF validation, GSE115574, which includes samples from 15 AF patients and 15 individuals with sinus rhythm, was employed. Gene expression values were retrieved using the GEOquery package in R, and differential expression of hub genes was confirmed across these datasets.

### Exploratory verification using clinical samples

Clinical serum samples were tested to corroborate the computational findings further. Twenty-three blood samples were obtained from twenty-three HCM patients with AF, in addition to samples from sixteen HCM patients without AF and ten healthy controls. The diagnosis of HCM was established by the observation of increased left ventricular wall thickness (≥ 15 mm) on imaging, which was not solely explained by abnormal loading conditions [13], while AF was confirmed by 12-lead electrocardiography. AF burden was defined as the percentage of total monitoring time during which the patient was in AF, as assessed by 24-hour ambulatory (Holter) ECG monitoring. The HCM-only and healthy control groups were aligned with the HCM + AF group regarding age and sex. All specimens were obtained at the First Affiliated Hospital of Harbin Medical University in Harbin, China. After collection, blood was centrifuged at 1000 × g for 10 minutes. Measuring the bioactive form of atrial natriuretic peptide (ANP) can be challenging due to its short half-life (2 minutes); therefore, we chose the more stable N-terminal fragment of A-type natriuretic peptide (NT-proANP), which has a significantly longer half-life in circulation. NT-proANP serum levels, reflecting the *NPPA* gene product, were quantified utilizing a human ELISA kit (Abcam, Shanghai, China). Participant recruitment and serum collection were conducted between June 20, 2025 and August 5, 2025.

### Statistical evaluation

All statistical analyses were performed using GraphPad Prism software (version 8.0.2). Continuous variables are presented as the mean ± standard deviation (SD) or as the median (interquartile range), depending on their distribution. Categorical variables are presented as frequencies and percentages (%). For continuous variables, comparisons between two independent groups were performed using the nonparametric Mann-Whitney U test, as the data did not meet the assumptions of normality. Comparisons among three independent groups were analyzed using the nonparametric Kruskal-Wallis test, followed by Dunn’s multiple comparisons test for post hoc analysis. Categorical variables were compared using the Chi-square test or Fisher’s exact test, as appropriate. A two-sided *P*-value < 0.05 was defined as statistically significant.

## Results

### Identification of differentially expressed and disease-associated genes

**Fig 1:**
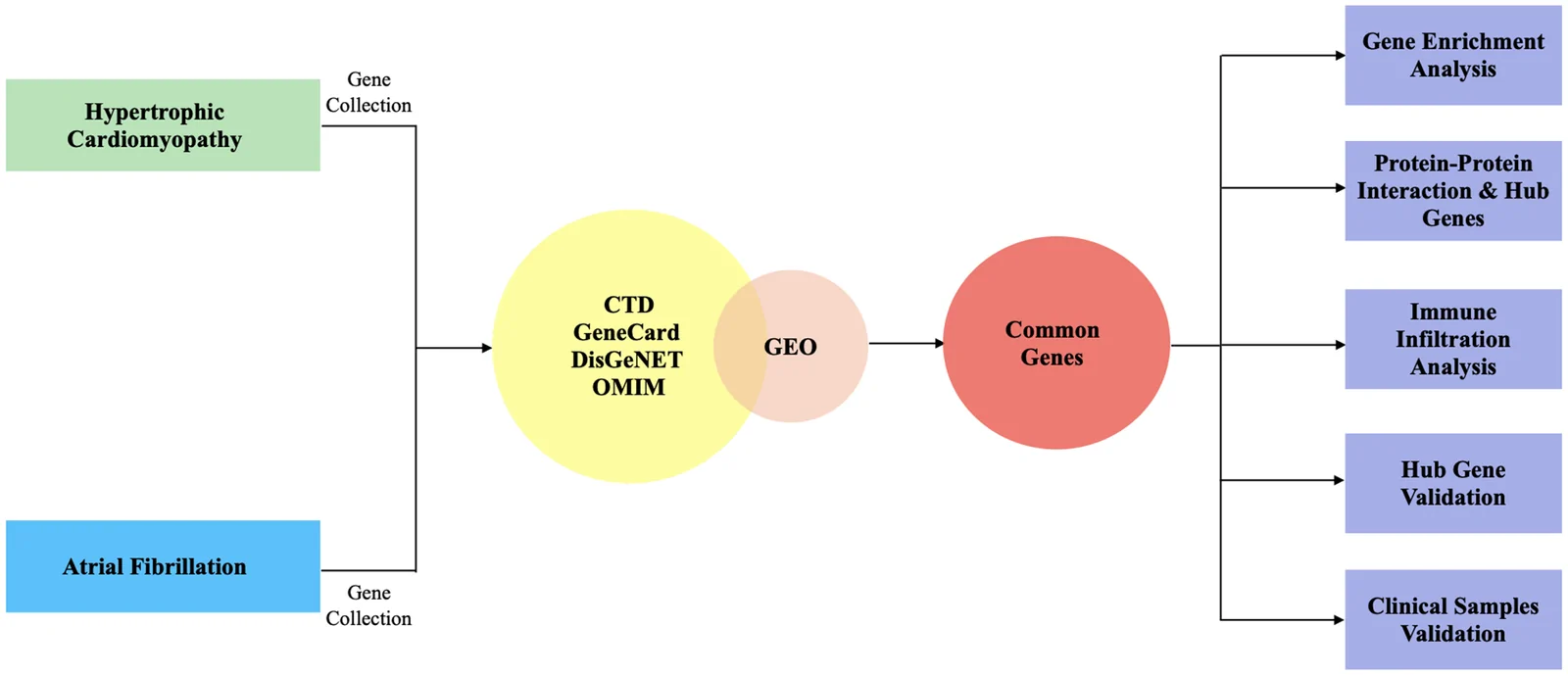
Overall study design and analytical workflow. The flowchart illustrates the systematic collection of genes associated with hypertrophic cardiomyopathy (HCM) and atrial fibrillation (AF) from multiple databases, followed by the identification of shared genes and subsequent downstream bioinformatics and clinical validation analyses.

A total of 1,054 DEGs were discovered from the GSE180313 dataset. Furthermore, 500, 123, 30, and 60 HCM-related genes were obtained from the GeneCards, DisGeNET, CTD, and OMIM databases. Following the consolidation and elimination of duplicates, and subsequently intersecting these genes with differentially expressed genes from GSE180313, 22 genes associated with HCM were identified (Fig 1).

**Fig 2:**
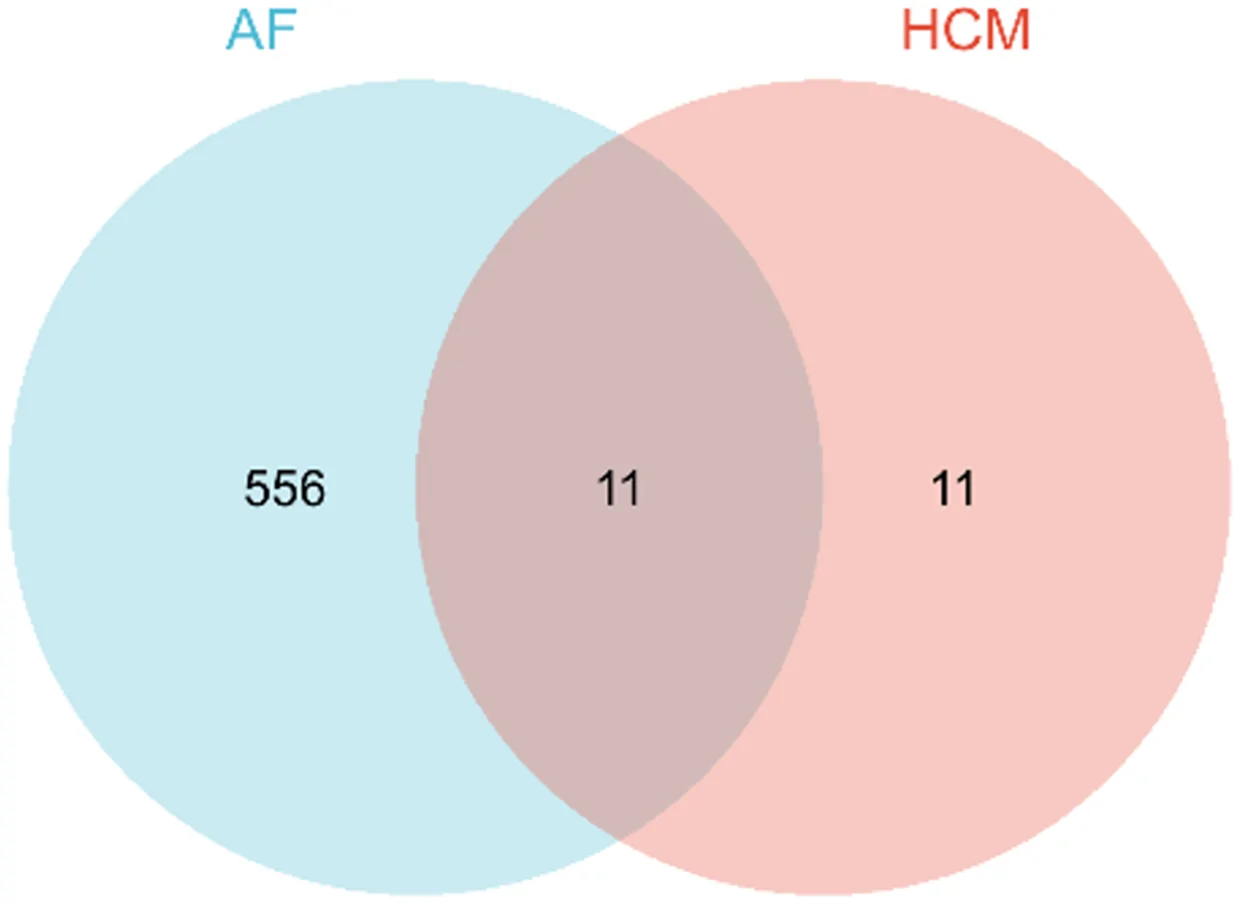
Venn diagram analysis reveals 11 genes shared between 567 AF-related genes and 22 HCM-related genes, suggesting common molecular pathways underlying both conditions. Abbreviation: HCM, hypertrophic cardiomyopathy; AF, atrial fibrillation.

For AF, 12,405 DEGs were obtained from the GSE41177 dataset and 126 from GSE79768, in addition to 500, 303, 500, and 55 AF-related genes sourced from the GeneCards, DisGeNET, CTD, and OMIM databases, respectively. The integration and deduplication of various sources resulted in 567 genes linked with AF. By intersecting the 22 genes associated with HCM and the 567 genes connected to AF, 11 genes were found as common to both HCM and AF. The comprehensive screening procedure and outcomes are illustrated in Table 1 and Fig 2.

**Table 1:** Collection of HCM and AF genes.

Disease	Database or GEO	Data Sources	Amount of Raw Data	Filter Condition	Amount of Data After Filtering and Deduplication	Merge	Common Genes
HCM	GEO	GSE180313	17337	If the raw data are greater than 500, then 500	1054	22	11
	Database	GeneCards	4973	are taken; if the raw data are less than 500, then all are included.	500		
		DisGeNET	158		123		
		CTD	30		30		
		OMIM	197		60		
AF	GEO	GSE41177	22185		12405		
		GSE79768	45118		126		
	Database	GeneCards	3588	FDR < 0.05 and |log2FoldChange|>1	500	567	
		DisGeNET	321		303		
		CTD	25764		500		
		OMIM	144		55		

Abbreviations: HCM, hypertrophic cardiomyopathy; AF, atrial fibrillation; FDR, false discovery rate.

### Gene enrichment analysis

**Fig 3:**
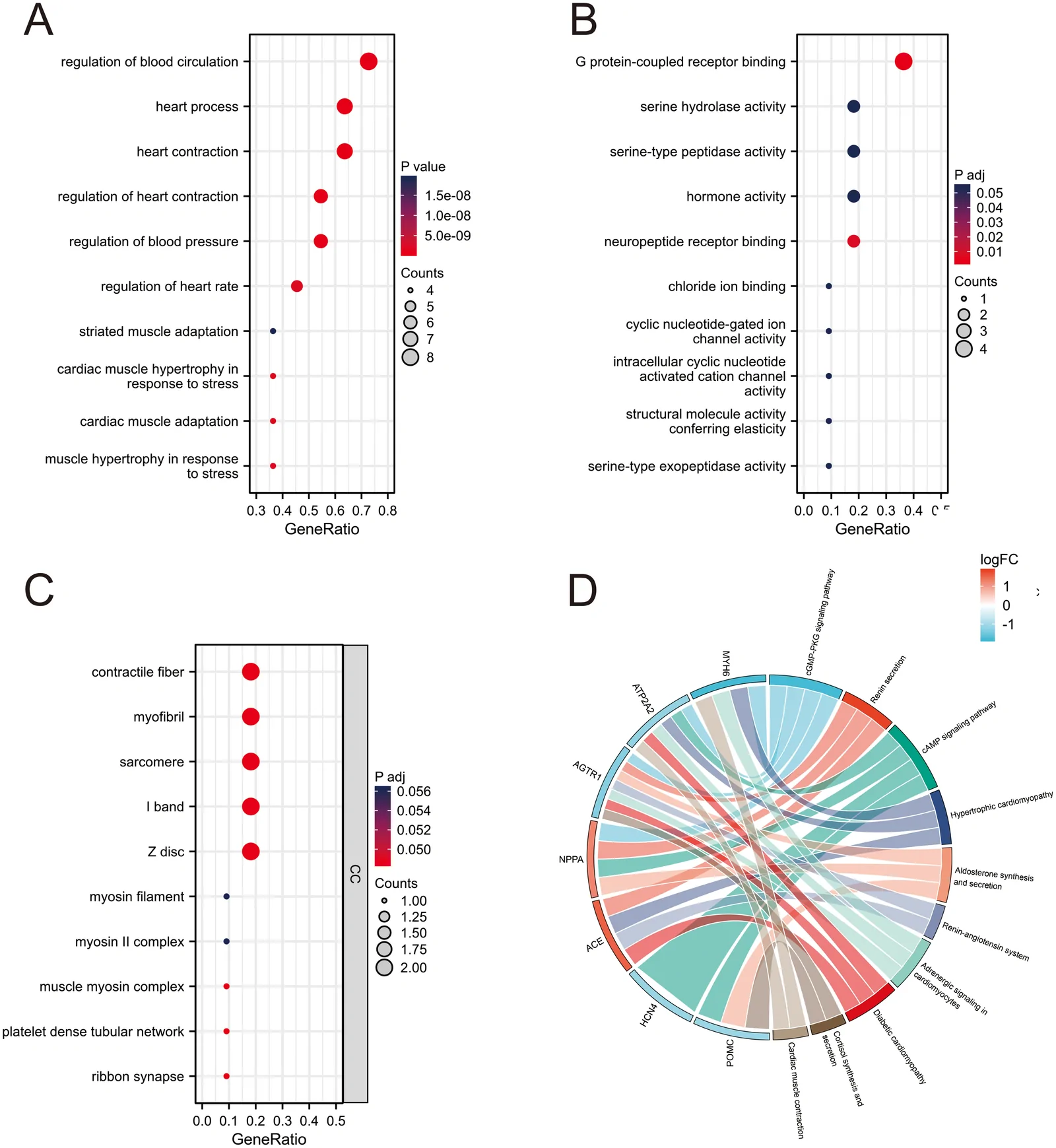
Functional enrichment analysis of the 11 shared genes: (A–C) Gene Ontology analysis highlights their involvement in key biological processes, molecular functions, and cellular components. (D) KEGG pathway enrichment identifies relevant signaling pathways that may link HCM and AF pathophysiology. Abbreviations: HCM, hypertrophic cardiomyopathy; AF, Atrial fibrillation; GO, Gene Ontology; KEGG, Kyoto Encyclopedia of Genes and Genomes.

The top 10 GO and KEGG pathways enriched among the 11 common genes are summarized in Fig 3. The genes showed a significant enrichment in biological processes associated with the regulation of blood circulation (Fig 3A). In the cellular component category, the primary association was with the sarcomere (Fig 3B), whereas the molecular function category emphasized G protein–coupled receptor binding (Fig 3C). Pathway enrichment analysis revealed significant connections with the calcium signaling pathway, dilated cardiomyopathy, and cardiac muscle contraction (Fig 3D).

### Network construction and prioritization of key hub genes

**Fig 4:**
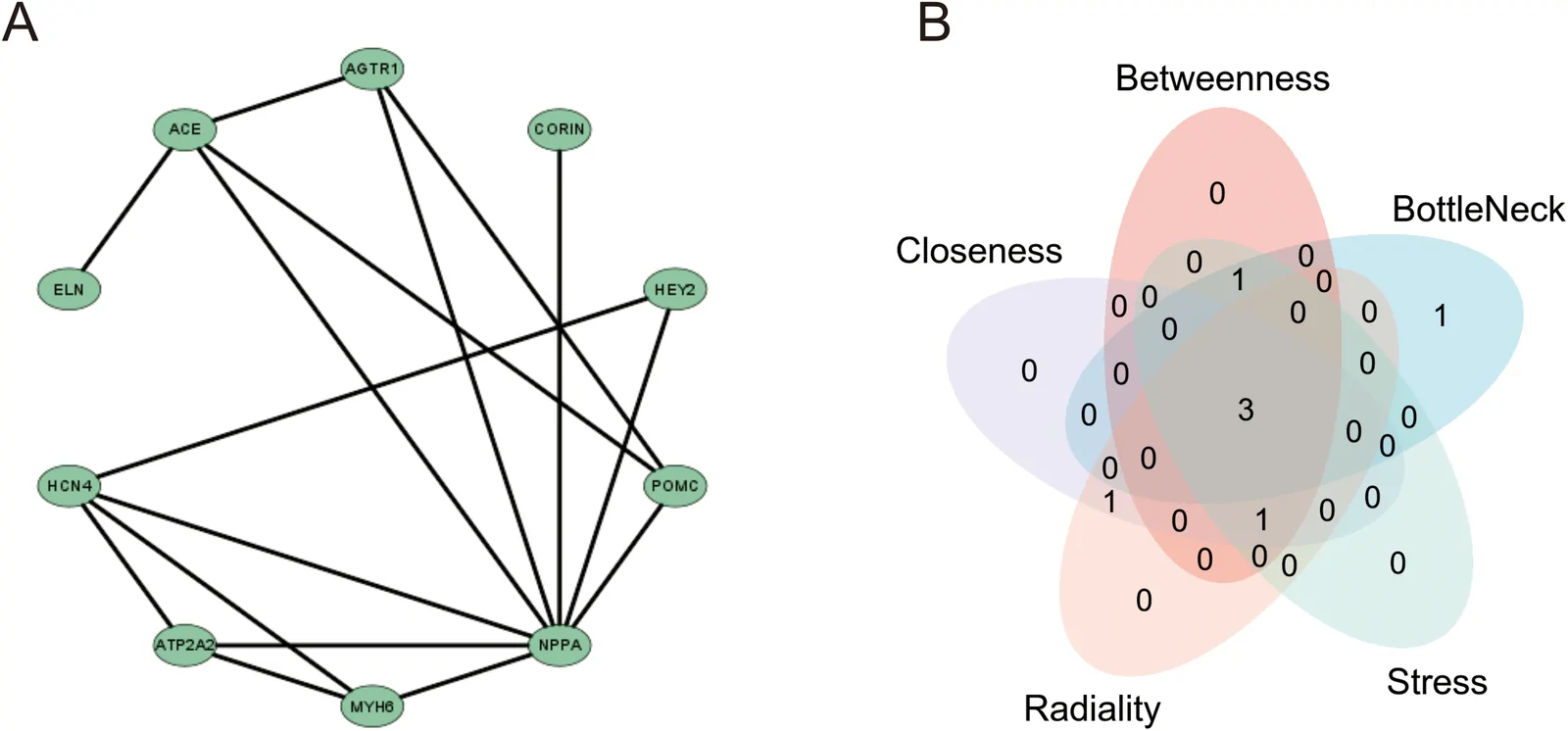
Protein–protein interaction network of the 11 shared genes: (A) Illustrates their interconnections. (B) *NPPA*, *ACE*, and *POMC* were identified as hub genes using five cytoHubba algorithms, indicating central roles in disease-related networks.

A PPI network was constructed utilizing the STRING database to investigate the interactions among the shared genes, applying a medium confidence threshold of > 0.4. The network produced comprised 11 nodes and 16 edges, and it was visualized with the aid of Cytoscape software (Fig 4A). Five computational algorithms: BottleNeck, Closeness, Radiality, Betweenness, and Stress were used within the CytoHubba plugin to rank the nodes. The convergence of the highest-ranked genes from these algorithms revealed three key hub genes: *NPPA*, *ACE*, and *POMC* (Fig 4B). In the analysis, *NPPA* and *ACE* exhibited upregulation in GSE180313, whereas *POMC* showed downregulation (Table 2).

**Table 2:** Hub genes.

Genes	BottleNeck	Closeness	Radiality	Betweenness	Stress	LogFC (GSE180313)
NPPA	10.00	8.50	3.33	50.00	52.00	1.22
ACE	2.00	6.50	2.89	16.00	16.00	1.57
POMC	1.00	6.00	2.78	0.00	0.00	−1.10

Abbreviations: LogFC, log_2_FoldChange; *NPPA*, Natriuretic peptide A; *ACE*, Angiotensin I converting enzyme; *POMC*, Proopiomelanocortin.

### Immune landscape profiling and hub gene correlation analysis

**Fig 5:**
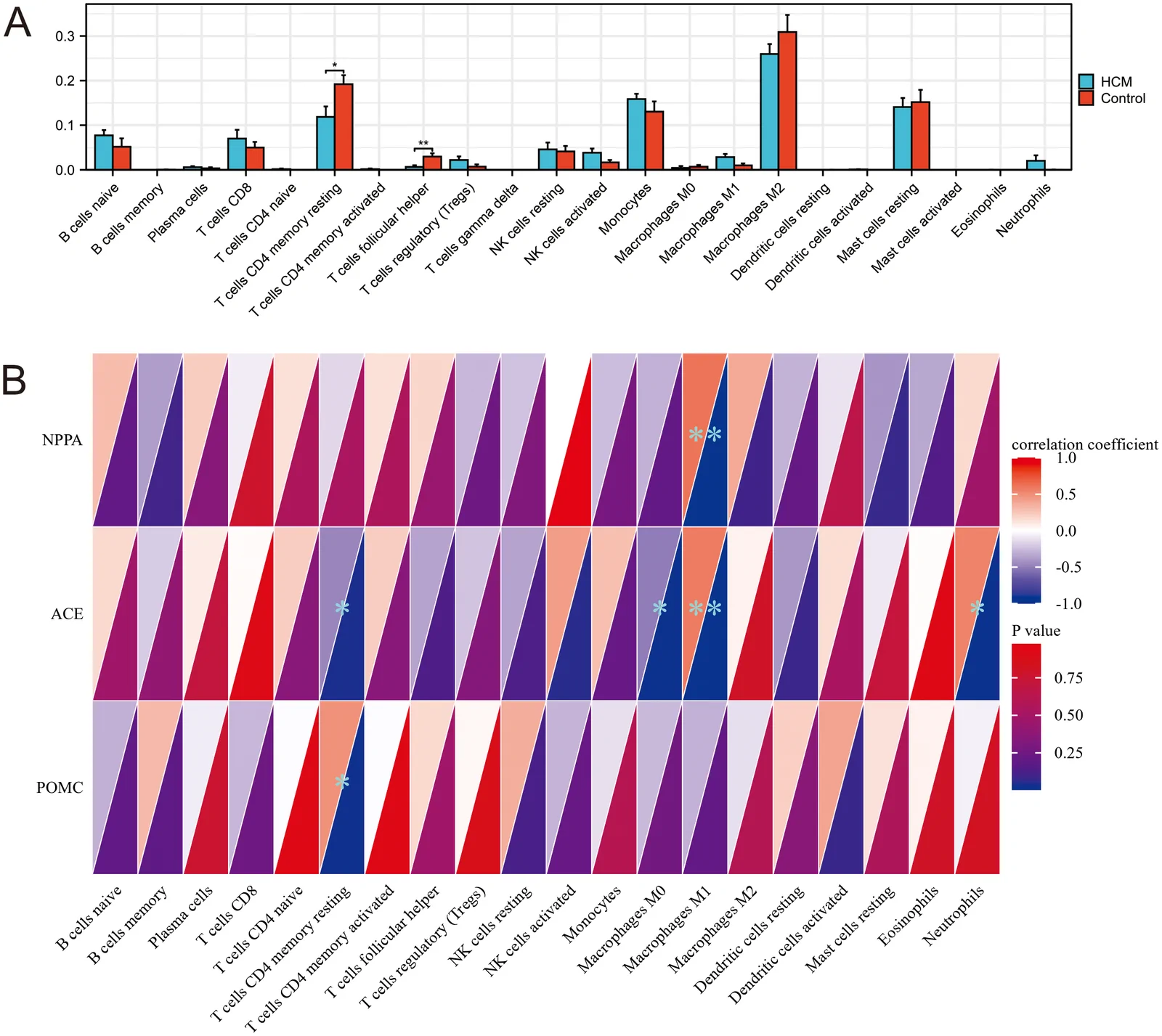
Immune cell infiltration analysis in HCM samples: (A) Shows the relative proportions of 22 immune cell types. (B) Correlation analysis demonstrates significant associations between hub genes and immune cell abundance (*P < 0.05; **P < 0.01), highlighting potential immunological involvement.

Evidence indicates that immune cell infiltration plays a significant role in the pathophysiology of HCM [14]. The CIBERSORTx analytical tool was employed to quantify the relative abundance of 22 immune cell types in the HCM expression dataset (Fig 5A). The immune landscape was primarily defined by the presence of B cells, CD8 ⁺ T cells, resting memory CD4 ⁺ T cells, follicular helper T cells, NK cells, monocytes, M2 macrophages, and resting mast cells. We conducted correlation analyses between gene expression levels and immune cell proportions to further investigate the immunological significance of the identified hub genes (Fig 5B). The expression of *NPPA* demonstrated a positive correlation with M1 macrophages. *ACE* expression was correlated with resting memory CD4 ⁺ T cells, M0 macrophages, M1 macrophages, and neutrophils, whereas *POMC* showed a relationship mainly with resting memory CD4 ⁺ T cells. The findings suggest that *NPPA*, *ACE*, and *POMC* may affect the immune microenvironment in HCM by interacting with macrophage and T cell populations.

### Cross-dataset validation of candidate hub genes

**Fig 6:**
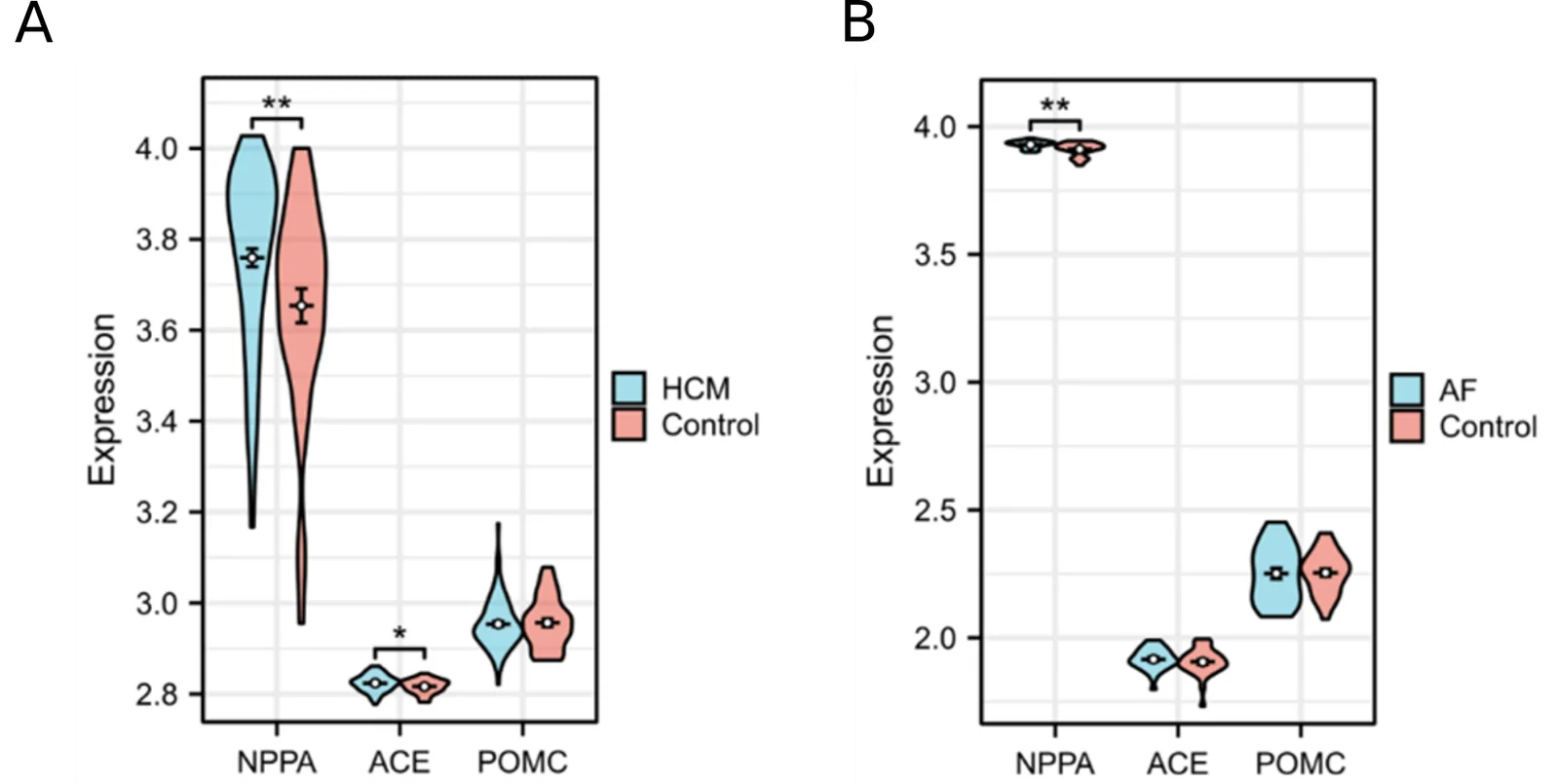
Validation of hub gene expression in external datasets: (A) *NPPA*, *ACE*, and *POMC* expression in the HCM dataset (GSE36961) and (B) in the AF dataset (GSE115574) confirms *NPPA's* consistent upregulation. (*P < 0.05; **P < 0.01).

The expression patterns of the identified hub genes were assessed in independent GEO datasets to confirm their reliability. The GSE36961 dataset revealed that *NPPA* and *ACE* exhibited markedly elevated expression levels in patients with HCM when contrasted with the control group (Fig 6A). Conversely, the GSE115574 dataset revealed that *NPPA* exhibited notable upregulation in patients with AF when compared to those in sinus rhythm.

### Exploratory clinical verification through serum NT-proANP quantification

**Fig 7:**
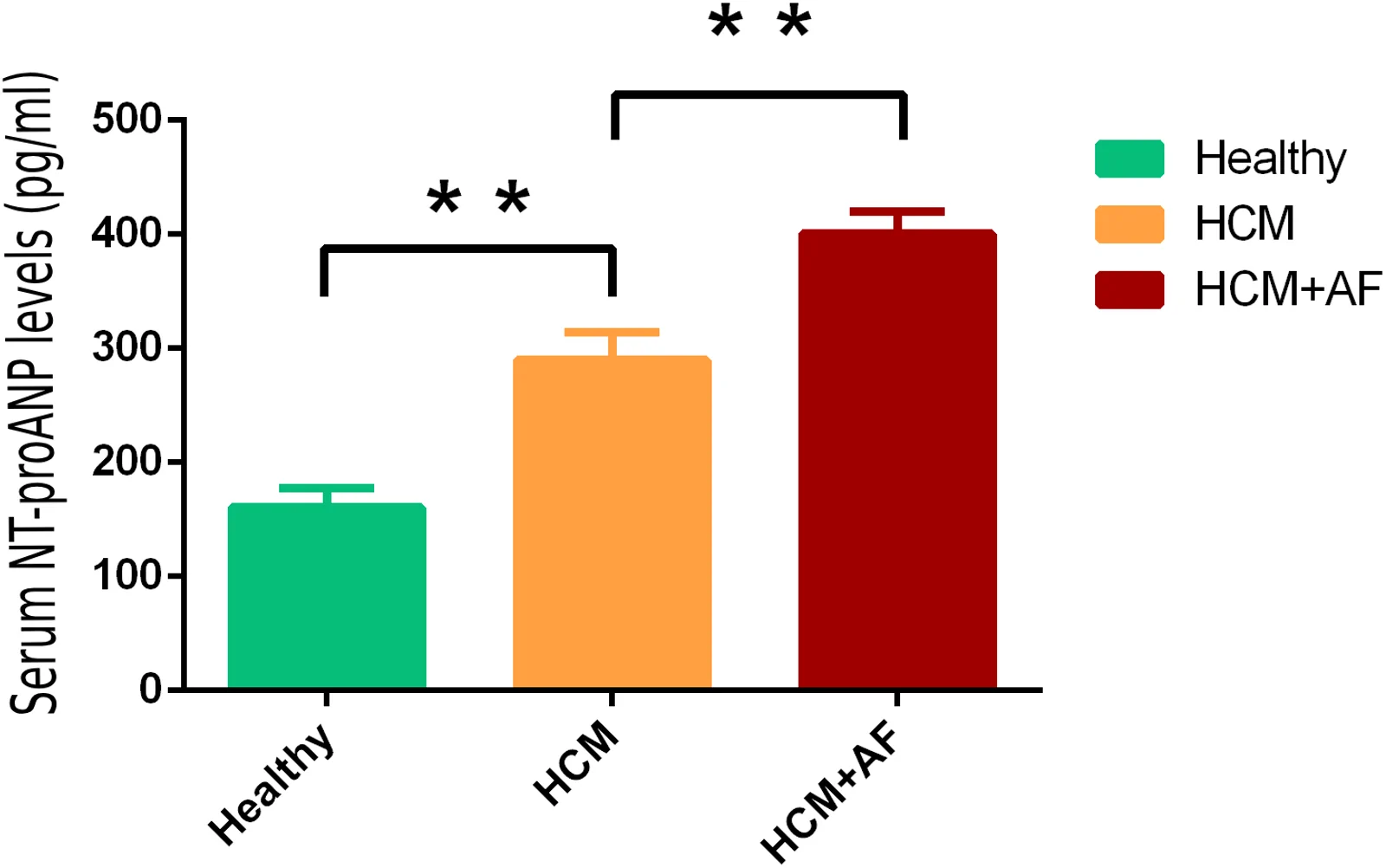
Serum NT-proANP levels measured in human samples (10 healthy controls, 16 HCM patients, 23 HCM patients with AF) demonstrate significant increases in disease groups (**P < 0.01), indicating potential clinical utility as a biomarker.

We investigated the expression of NT-proANP, the protein product of *NPPA*, in human serum samples to validate *NPPA*’s role as a pivotal gene linking HCM and AF. Serum NT-proANP levels were assessed in ten healthy controls, sixteen patients with HCM and twenty-three patients with HCM and AF. The baseline clinical and echocardiographic characteristics of the study cohort are summarized in Table 3. Fig 7 illustrates that NT-proANP concentrations were significantly higher in both the HCM and HCM + AF groups compared to healthy individuals (*P* < 0.01). The HCM + AF group demonstrated significantly elevated NT-proANP levels compared to the HCM-only group (*P* < 0.01).

**Table 3:** Echocardiographic and clinical variables of HCM and HCM with AF.

Variable	HCM (*n* = 16)	HCM with AF (*n* = 23)	*P*-value
LA diameter, mm	42.0 (40.5, 43.0)	43.0 (41.5, 44.0)	0.137
E/e’ Ratio	12.0 (9.5, 14.5)	13.2(10.8, 16.0)	0.138
Systolic anterior motion of MV, n (%)	3(18.8%)	5(21.7%)	1.00
Mitral Regurgitation, n (%)	4(25.0%)	7(30.4%)	1.00
Age	64 (60.0, 68.0)	61(56.5, 66.5)	0.391
Male, n (%)	10(62.5%)	9(39.1%)	0.200
Serum NT-proANP, pg/mL	293.8 (275.7, 307.9)	395.7 (389.5, 408.0)	< 0.01
NT-proBNP, pg/mL	168.3 (160.0, 185.3)	176.2 (165.4, 196.3)	0.236
Creatinine, μmol/L	78 (70, 85)	82 (75, 90)	0.220
AF Burden, %	0	36.7 (34.9, 39.1)	N/A

Abbreviations: AF, atrial fibrillation; LA,left atrium; MV, mitral valve; NT-proANP, N-terminal fragment of A-type natriuretic peptide; NT-proBNP, N-terminal fragment of brain natriuretic peptide. *P*-values were calculated using the Mann-Whitney U test for continuous variables and Fisher’s exact test for categorical variables.

## Discussion

Numerous studies indicate a strong association between HCM and the development of AF, which significantly increases mortality and complications in patients with HCM [15]. The pathogenesis of susceptibility to AF in HCM remains incompletely elucidated. A genetic predisposition to AF has been suggested, given its heightened occurrence in certain families affected by HCM. Indeed, this phenomenon cannot be exclusively attributed to hemodynamic factors [16,17]. Genetic disorders have been demonstrated to directly increase the susceptibility to primary atrial cardiomyopathy, and consequently to AF [18,19]. In this study, we first performed a genomic analysis of HCM and AF to identify genetic associations between the two diseases.

We discovered 11 common genes shared by HCM and AF. Enrichment analysis of the 11 common genes suggested involvement in pathways regulating blood circulation. Given the recognized role of inflammation in cardiovascular disease, we performed an exploratory immune profiling of ventricular myocardial tissue from the HCM cohort (GSE180313 dataset) using CIBERSORT. Given that the ventricular myocardial tissue data may not directly represent the atrial substrate for AF, this immune profile analysis is presented as a hypothesis-generating exploration of the shared inflammatory milieu in HCM and AF hearts. Increasing research suggests that communication between immune cells and cardiomyocytes is also vital to the pathophysiology of cardiac hypertrophy [20]. The analysis revealed significantly reduced infiltration levels of resting memory CD4 T cells and T follicular helper cells in HCM myocardial tissues compared to healthy controls, consistent with a prior report by Gong et al. [14], suggesting a potential immune dysregulation role in HCM pathogenesis. Resting memory CD4 T cells are typically in a quiescent state but retain antigen-specific memory to mount rapid responses upon re-exposure. Their reduced infiltration observed in HCM could indicate an altered immune microenvironment that promotes pro-inflammatory responses. This altered state may potentially facilitate pro-inflammatory signaling pathways associated with hypertrophy, such as mitogen-activated protein kinase (MAPK) and phosphoinositide 3-kinase (PI3K)-Akt [14]. The role of the similarly reduced T follicular helper cells in HCM is less clear, though their decrease might also contribute to an overall immune imbalance. These findings in ventricular tissue suggest that immune dysregulation is a feature of HCM. Future experimental investigations, both in vitro and in vivo, are essential to determine if and how these specific immune cell changes directly drive atrial remodeling and AF pathogenesis in HCM.

Later, a comprehensive analysis was conducted to construct the PPI network, demonstrating genetic and protein-based relationships among common genes shared between HCM and AF. *NPPA*, *ACE*, and *POMC* were identified as hub genes, with *NPPA* confirmed through external datasets and human samples. The findings indicate that *NPPA* is a significant gene associated with both HCM and AF. The *NPPA* gene, which encodes ANP, is predominantly expressed in the atria [21]. One of the most effective triggers for ANP expression and release is mechanical stretching of the atrial wall, not LV pressure gradients or ventricular mass [22–24]. Previous studies have shown that plasma ANP levels increased with left atrial dysfunction [25]. In contrast, elevated plasma BNP in HCM primarily reflects LV obstruction and diastolic dysfunction [22,26]. The diagnostic role of circulating ANP in HF has been extensively described. However, it is unclear whether ANP plays a role in predicting AF in HCM. Studies have shown that high plasma ANP levels are associated with an increased risk of AF in a male population [27]. This study observed a significant upregulation of *NPPA*/ANP expression in AF patients, a finding consistent with a previous report [28]. Our results support the interpretation that *NPPA*/ANP serves as an indicator of atrial electrical remodeling in HCM rather than an initiating factor in AF development. In HCM, diastolic dysfunction and increased ventricular stiffness elevate LA pressure to maintain ventricular filling. This chronic pressure overload causes LA dilation and mechanical stretch of atrial myocytes, resulting in electrical remodeling and the development of AF. However, our study identifies a distinct biomarker signature in HCM patients who develop atrial fibrillation. NT-proANP levels were significantly elevated in the HCM + AF group relative to patients with HCM alone. Notably, this difference occurred without concomitant changes in key conventional parameters, including LA diameter, the ventricular stretch marker N-terminal fragment of brain natriuretic peptide (NT-proBNP), and the echocardiographic index of LV diastolic function (E/e’). The isolated increase in NT-proANP suggests compartmentalized atrial remodeling that may precede ventricular dysfunction. Recent genomic studies have revealed a complex genetic architecture underlying AF susceptibility [29]. Clinically, this means that in genetically predisposed individuals, AF may begin to develop before overt hemodynamic alterations or structural changes in the left ventricle become apparent, a pathophysiology reflected in the new concept of lone AF [30]. Our findings support the hypothesis that elevated ANP is a more sensitive biomarker of the atrial proarrhythmic substrate in HCM, potentially reflecting early atrial-specific electrical remodeling before the onset of atrial dilation. These preliminary findings require confirmation in larger, prospective cohort studies to validate the clinical utility of ANP as a biomarker for AF risk stratification in HCM.

## Limitations of the study

This study has several limitations. The number of clinical samples analyzed was relatively small, which may limit the generalizability of the results. Furthermore, the observed association between ANP and AF could be influenced by unmeasured confounders related to disease severity or hemodynamic status, which our study was not powered to adjust for. Although the results consistently highlight *NPPA* as a key gene linking HCM and AF, larger cohorts will be required in future studies to confirm these observations and strengthen statistical power.

## Conclusion

In this study, we identified 11 differentially expressed genes shared between HCM and AF and constructed a co‑expression network linking the two conditions. Clinical validation analyses highlighted *NPPA* as a candidate hub gene implicated in both disorders, and its upregulation was significantly associated with incident AF in the HCM cohort. Notably, its encoded peptide, NT‑proANP, represents a promising predictive biomarker for identifying HCM patients at increased risk of developing AF. Further mechanistic investigations and large‑scale longitudinal studies are warranted to verify these associations and establish its clinical predictive utility.

## Abbreviations

*ACE*: Angiotensin I converting enzyme
AF: atrial fibrillation
ANP: atrial natriuretic peptide
BNP: brain natriuretic peptide
CTD: Comparative Toxicogenomics Database
DEGs: differentially expressed genes
ECG: electrocardiogram
ELISA: enzyme-linked immunosorbent assay
FDR: false discovery rate
GEO: Gene Expression Omnibus
GO: Gene Ontology
HCM: hypertrophic cardiomyopathy
HF: heart failure
KEGG: Kyoto Encyclopedia of Genes and Genomes
LA: left atrium
LogFC: log2FoldChange
LV: left ventricular
LVOTO: left ventricular outflow tract obstruction
MAPK: mitogen-activated protein kinase
MV: mitral valve
NK: natural killer
*NPPA*: Natriuretic peptide A
NT-proANP: N-terminal fragment of A-type natriuretic peptide
NT-proBNP: N-terminal fragment of brain natriuretic peptide
OMIM: Online Mendelian Inheritance in Man
PI3K: phosphoinositide 3-kinase
*POMC*: Proopiomelanocortin
PPI: protein-protein interaction
SD: standard deviation
